# Protective Effects of N-Acetyl-L-Cysteine on the Density of Spiral Ganglion Cells and Histological Changes Induced by Continuous Noise Exposure in Rats

**DOI:** 10.21315/mjms2018.25.5.5

**Published:** 2018-10-30

**Authors:** Raheleh Hashemi Habybabady, Seyed Bagher Mortazavi, Ali Khavanin, Ramazan Mirzaei, Mohammad Reza Arab, Behzad Mesbahzadeh, Mehran Hoseini, Mahdi Mohammadi

**Affiliations:** 1 Health Promotion Research Center, Department of Occupational Health Engineering, Zahedan University of Medical Sciences, Zahedan, Iran; 2 Department of Occupational Health Engineering, School of Medical Sciences, Tarbiat Modares University, Tehran, Iran; 3 Department of Occupational Health Engineering, School of Health, Mashhad University of Medical Sciences, Mashhad, Iran; 4 Cell and Molecular Research Center, Department of Anatomical Sciences, Zahedan University of Medical Sciences, Zahedan, Iran; 5 Department of Physiology and Pharmacology, Birjand University of Medical Sciences, Birjand, Iran; 6 Expert of Public Health, Birjand University of Medical Sciences, Birjand, Iran; 7 Health Promotion Research Center, Department of Biostatistics & Epidemiology, Zahedan University of Medical Sciences, Zahedan, Iran

**Keywords:** N-acetyl-l-cysteine, noise, cochlea, histology, spiral ganglion cells, hearing loss

## Abstract

**Background:**

Noise exposure causes loss of cochlea hair cells, leading to permanent sensorineural hearing loss, and initiates pathological changes to the bipolar primary auditory neurons (ANs). This study focuses on the effects of N-acetyl-l-cysteine (NAC) in protecting the density of spiral ganglion cells and in histological changes induced by continuous noise exposure in rats.

**Methods:**

Twenty-four male Wistar rats were randomly allocated into four experimental groups to receive NAC, saline, noise, or both noise and NAC. Noise exposure continued for ten days. Saline and NAC were injected daily during the noise exposure, and 2 days before and after the noise exposure. Evaluation of cochlear histopathology and the density of spiral ganglion cells was performed 21 days after exposure.

**Results:**

In the animals exposed to noise, a reduction in the density of spiral ganglion cells was evident in both the basal and middle turns of the cochlea. This improved on receiving NAC treatment (*P* = 0.046). In the histopathology evaluation, some histological changes, such as disorganised architecture of the outer hair and supporting cells and a slightly thickened basilar membrane, were found in the basal turns in the noise group.

**Conclusion:**

NAC offered partial protection against noise exposure by improving the density of spiral ganglion cells and reducing morphological changes.

## Introduction

The prevalence of hearing loss is approximately 10% worldwide, and half of auditory damage is related to intense noise exposure (1). One of the identified mechanisms underlying noise-induced hearing loss (NIHL) is oxidative damage in the inner ear caused by excessive formation of free radical species, including reactive oxygen species (ROS) and reactive nitrogen species (RNS) (2). ROS directly damage all components of cells by adversely altering proteins, lipids, and DNA; they also indirectly affect genes involved in apoptosis (3–6). Noise exposure causes loss of cochlea hair cells, leading to permanent sensorineural hearing loss (SNHL), and initiates pathological changes to the bipolar primary auditory neurons (ANs). Unmyelinated peripheral processes are rapidly and extensively reduced within the organ of Corti (OC), which normally innervates the inner hair cells (IHCs). The myelinated portion of the peripheral processes is gradually degenerated, resulting in cell death. Although ANs are anatomically different in humans and animals, the human cochlea is also subject to this general neural degeneration pattern, albeit at a much slower rate (7).

Although there are no reported findings on loss of spiral ganglion cells (SGCs) in rats exposed to noise (6), some studies indicate that the direct effects of noise include immediate swelling and rupturing of the nerve terminals, even in cases with no hair cell death. Acoustic over exposure can cause death of SGCs even if IHCs survive (6, 8, 9). In addition to damage to SGCs, noise can lead to histological changes such as degeneration, vacuolation, and disruption in hair cells, supporting cells, and pillar cells, and degeneration of fibrocytes of the spiral ligament with psychotic nuclei (9).

Recently, researchers have focused on developing new free radical scavengers for greater protection of the inner ear and neural fibres against noise exposure (9). N-acetyl-l-cysteine (NAC) is a thiol-containing amino acid derivative that acts as an ROS scavenger and as a substrate for production of glutathione, the major endogenous antioxidant produced by cells. NAC has been shown to reduce NIHL and can protect functional changes in hearing in animal models (4, 10, 11). Although NAC has appeared promising in some studies, other studies have found that it had no effect in exposure to continuous broadband noise (12, 13).

Although previous studies have assessed the effect of anti-oxidants on noise-induced cochlear damage, their experimental designs were different in terms of animal species, spectrum of noise frequency, noise intensity, and duration of exposure; they also assessed NIHL at various time periods after exposure using diverse assessment techniques (6, 9, 14–17). Most studies have focused on functional changes in hearing after noise exposure (10, 18), and there is little evidence about the effect of NAC on noise-induced SGCs and histological changes in hearing (16). As the nature of histological changes due to NAC treatment in the inner ear is unclear, and as histological studies help to select appropriate functional tests and pharmaceutical treatment for SNHL, this study was designed to evaluate the protective effect of NAC on the density of SGCs and on histological changes induced by continuous white noise exposure in rats.

## Materials and Methods

### Animals

In this experimental study, a total of 24 male Wistar rats with an average weight of 275 g (ranging between 250 g and 300 g) and no evidence of middle ear infection were obtained from the Animal Research Centre of Zahedan University of Medical Sciences (Iran). Food (rodent chow, Pars Animal Co., Iran) and water were available ad libitum, except during the experiment. Lighting was on between 07:00 and 19:00. Animals were kept at a temperature of 21 °C (ranging between 20 °C and 22 °C), with humidity ranging between 40% and 50% (19).

### Sample Size

A total of 24 male rats with normal Preyer reflex were allocated equally and at random, based on a permuted-block design, to four experimental groups of six rats: saline treatment only (group name: Sham), noise trauma only (group name: Noise), noise trauma and NAC treatment (group name: NoiseNAC), and NAC treatment only (group name: NAC).

According to the pilot study, the SGC density in the Sham group had a standard deviation (SD) of 6. Assuming an expected mean difference of 12 between the NoiseNAC and Sham groups, a sample size of six animals was taken for each group, with α and β as 0.05 and 0.1, respectively, and a missing fraction of 20%.

### Noise Exposure

A ventilated reverberant chamber with dimensions of approximately 60 × 45 × 30 cm^3^ was constructed for the experiment. The primary design took into account all the considerations proposed in previous studies (20–23). On each experimental day, each of the six animals was moved into an individual wire mesh cage (17 × 13 × 15 cm^3^). Animals in the NoiseNAC and Noise groups were exposed to 102 dB SPL (ranging between 101.5 and 102.5 dB SPL) high-pass white noise centred at 8 kHz, for 8 h a day over 10 consecutive days in the exposure chamber. In order to eliminate environmental factors, animals in the Sham and NAC groups were placed in the same conditions inside the ventilated reverberant chamber for 10 days without noise exposure.

Filtered noise generator software (Timo Esser’s Audio software, version 1.2) generated the noise, which was then delivered using Cool Edit Pro 2.1 (Syntrillium Software Corporation), amplified with an amplifier (Rock Jw-s317, China), and propagated by four loudspeakers (Microlab HT 25 Tweeter, Italy) located 0.15 metres above the cages inside the chamber. A sound level meter (CEL-450 type 1D, Casella CEL) equipped with an analyser was used to monitor the sound level inside the chamber. At each side of the chamber at the animal’s head level, a hole was made and tapped to measure the sound level within the chamber during the experiment, thus ensuring the uniformity of the stimulus (with a maximum variation of 0.5 dB).

### Drug Treatment

**Figure 1:**
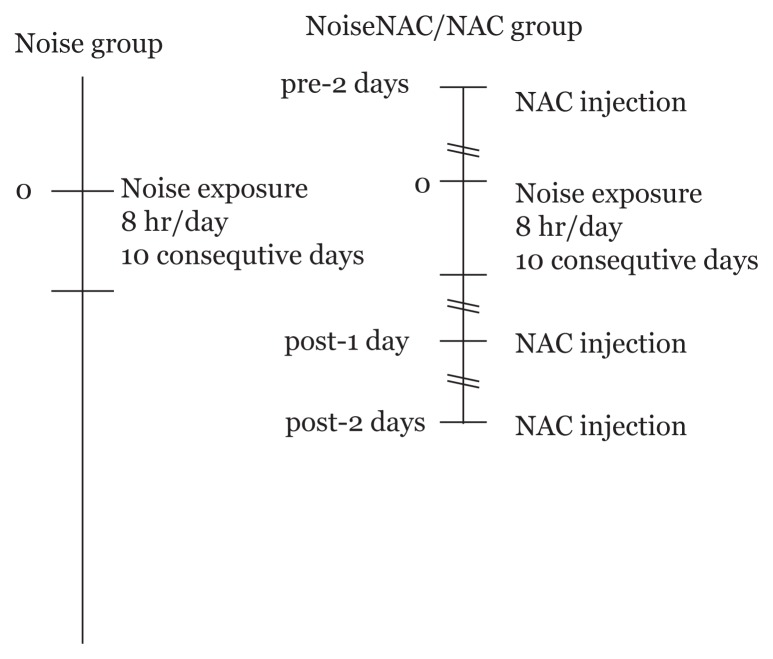
Schematic experimental protocol of noise exposure and NAC injections

NAC (Zambon SpA, Italy) at a dose of 325 mg/kg was injected intraperitoneally daily for 2 days prior to noise exposure, 1 h before exposure for 10 consecutive days, and on two days of post-noise treatment, giving a total of 14 injections. The Sham group was injected with a similar volume of normal saline over the same schedule as the NAC-treated group (Figure 1).

The NAC treatment protocol was based on the most effective protocols proposed by previous studies. It is already known that this dose of drug is not ototoxic and does not produce permanent hearing loss (15, 24, 25). Accordingly, the intraperitoneal injection of NAC was administered 2 days before the commencement of noise exposure, 1 h before each episode of exposure, and up to 48 h after exposure, in order to prevent hair cell loss (15, 24, 25). Due to the water-soluble properties of NAC and the delayed production of free radicals after exposure to noise, there is a therapeutic window of opportunity for injections of NAC to be effective in hearing protection (26).

### Histology

Temporary and permanent hearing loss was measured 21 days after the last exposure to noise and antioxidant injections (data not shown). Taking into account of the 12 days of exposure, the total duration of the experiment was therefore 33 days. Afterwards, all animals were anesthetised with a mixture of ketamine (68 mg/kg) and xylazine (8 mg/kg) and immediately transcardially perfused with cold 0.09% saline followed by a fixative solution containing 4 % paraformaldehyde (pH 7.4). Their temporal bones were then removed rapidly, the bulla opened, and the cochlea post-fixed in the same fixative for 48 h at 4 °C. Afterwards, the samples were decalcified by immersion in 0.1 mol/L ethylenediaminetetraacetic acid (EDTA) for one week. The decalcified tissues were processed using routine histological methods and embedded in paraffin blocks. The samples were cut on the perimodiolar plane (at a thickness of 5 μm) using a rotary microtome (Leitz, Italy). Every third section was mounted on a glass slide and stained with haematoxylin and eosin.

For each animal, five mid-modiolar sections were selected, including the lower basal, upper basal, lower middle, upper middle, and apical turns of the cochlea. For qualitative and quantitative analysis of OC and SGCs, images were taken using a light microscope (Olympus BX41, Melville, NY). The outline of the Rosenthal’s canal profile was then traced, and the SGCs were counted from the base to the apex of the cochlea within each profile in a double-blind manner using Image J software (version 1.44p, National Institute of Health, USA). The density of SGCs was expressed in an area of 10,000 μm^2^ (27, 28). The mean density in every turn was considered for each animal.

### Statistical Analysis

The density of SGCs was described as a mean (SD). The density data was confirmed to be normally distributed by using the Shapiro–Wilk test on each study group. Mean densities were compared using the one-way ANOVA and post-hoc Tukey HSD tests in SPSS version 18.0. The independent and dependent variables were the group and the density, respectively. The significance level was taken as 0.05.

### Ethical Considerations

According to the principles of the Helsinki Protocol, every effort was made to reduce animal suffering and the number of animals needed for the experiment. The study protocol was approved by the Ethics Committee for Experimental Medicine of Tarbiat Modares University (code 8619, March 2014).

## Results

**Figure 2:**
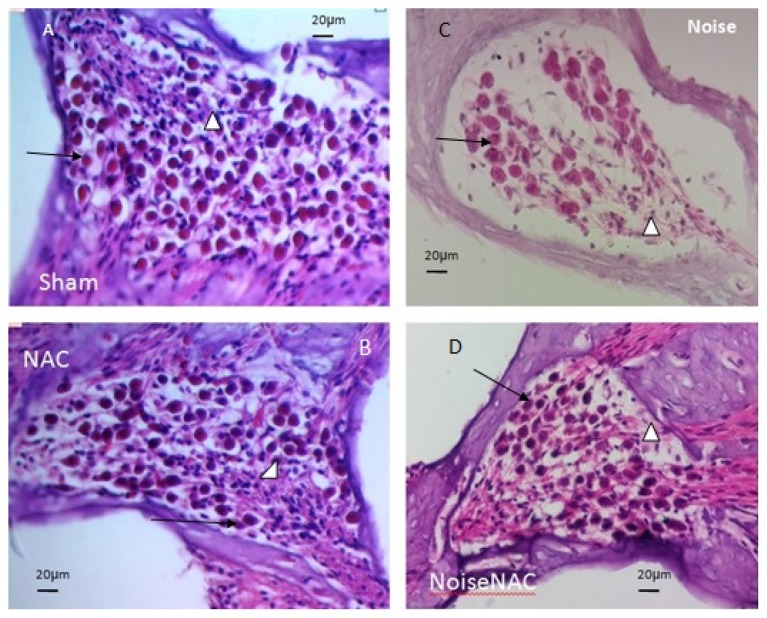
A–D show SGC density in histological examination in the Sham (A), NAC (B), Noise (C), and NoiseNAC (D) groups. In the Sham and NAC groups, Rosenthal’s canal was densely packed with SGCs and fascicles of auditory nerve fibres (A–B). In the Noise group, the density of SGCs was reduced considerably in the lower basal turn area (C). NAC administration in NoiseNAC group replenished reduced SGCs density after noise exposure (D). Arrows and arrowheads indicate SGCs and auditory fibres, respectively

In the Sham and NAC groups, Rosenthal’s canal was densely packed with SGCs and fascicles of auditory nerve fibres with eosinophilic cytoplasm and central vesicular nuclei (Figures 2A–B). The process of degeneration of SGCs was clearly observed using hyperchromasia, and displacement of the nucleus to the cellular periphery was evident 21 days after noise exposure. Extensive reduction in the density of SGCs occurred mainly in the lower and upper basal turns and, to a moderate extent, in the middle turns (Figure 2C). The number of preserved SGCs was increased by administration of NAC (Figure 2D).

The reduction in SGC density was greater in the Noise group than in the Sham group in both the basal and middle turns of the cochlea. The reduction in the NoiseNAC group was less than that in the Noise group. However, the NAC treatment did not provide full recovery in the Sham group. The most pronounced reduction of SGCs occurred in the lower basal turn in the Noise group, which was significantly different from that in the Sham and NAC groups (*P* < 0.001). In the lower basal turn, the density of SGC in the NoiseNAC group was significantly different from that of the Sham group (*P* < 0.001), the NAC group (*P* < 0.001), and the Noise group (*P* = 0.046) (Table 1).

**Figure 3:**
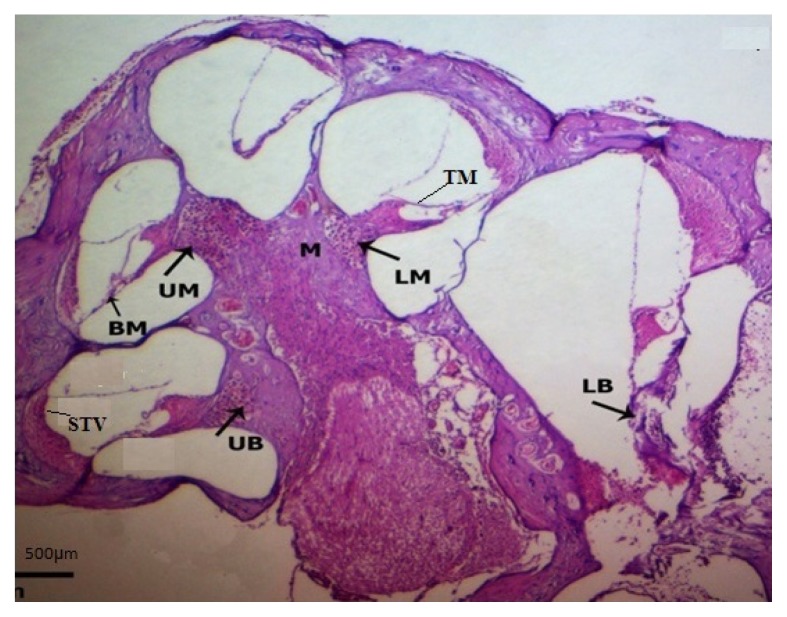
Histological examination showing appearance and histology of OC, TM, and STV in the lower basal (LB), upper basal (UB), lower middle (LM), upper middle (UM), and apical turns of the cochlea in the Sham and NAC groups

Histological examination of animals in the Sham group demonstrated normal appearance of the cochlear duct in the lower and upper basal turns. In the outer wall of the cochlear duct, the stria vascularis (STV), with its eosinophilic appearance of dark and light cells, was clearly observed. Three rows of outer hair cells (OHCs) with columnar appearance and eosinodophilic cytoplasm and vesicular nuclei were visible in the OC; a row of IHCs with similar morphology was also observed. The morphology of all the supporting cells was normal. The tectorial membrane (TM), with its tip turned upwards, was seen hanging as a homogenous eosinophilic structure on the apical surface of the cells. There was a special eosinophilic zone in the apical surface of the hair cells adjacent to the TM. In the middle turn, the appearance of the cochlear duct and the STV was similar to that in the basal turns, but the OHCs did not show a well-defined pattern, with cells varying in their positions. The TM membrane stretched towards the OHCs more than in the basal turns, with its tip turned upwards (Figure 3).

**Figure 4:**
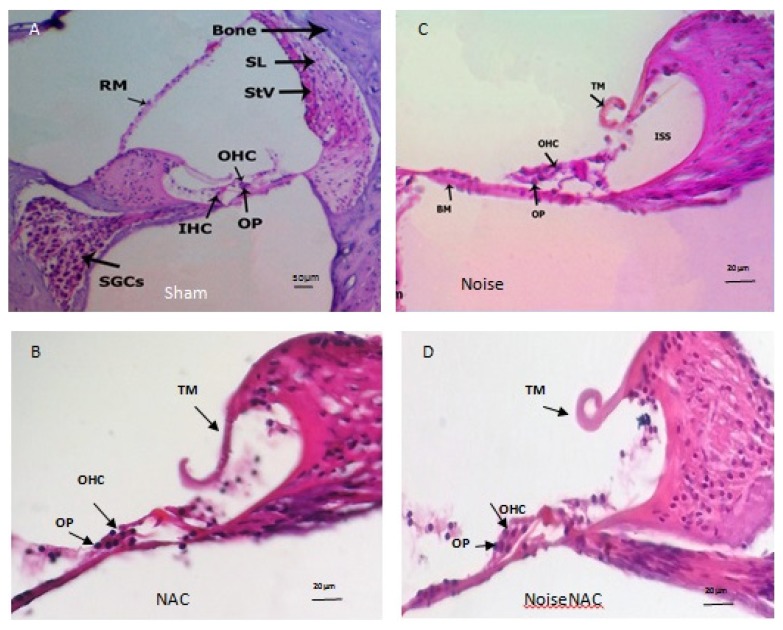
Histological examination showing normal appearance and histology of cochlea in the Sham and NAC groups (A–B), disorganised architecture of OHCs and outer phalangeal (OP) cells and disappearance of IHCs in the Noise group (C), decrease in TM height and normal appearance of OHCs, IHCs, and OPs in the NoiseNAC group (D)

Histological evaluation of the NAC group showed no histological difference in the cellular arrangement of the OC, including hair cells, supporting cells, and TM, compared to the Sham group (Figures 4A–B).

According to the histological examination of the Noise group, OHCs in the lower basal turn were slightly affected after noise exposure: their cell integrity and eosinophilic characterisation were lost, and the nuclei were small and pyknotic. It appears that the reduction in OHC height resulted in an increase in the distance between the apical surface of the OHCs and the TM. In the Noise group, the IHCs and supporting cells could not be recognised clearly. There was an increase in staining intensity in the basilar membrane, and more eosinophilic characterisation was observed in comparison to the Sham group. Additionally, a slight increase in the thickness of the STV was observed.

Disorganised architecture of OHCs and supporting cells was a prominent feature of the histological findings in the experimental Noise group, including nuclear stratification of OHCs and cellular displacement of hair cells and supporting elements. The results showed a slight shift in the arrangement of OHCs towards the distal end of the TM (Figure 4C). The morphological appearance in the OC, STV, TM, cochlear duct, and basal membrane in the middle and apical turns was similar to that of the Sham and NAC groups.

The reduction in the height of the TM and the disorganised architecture of IHCs in the NoiseNAC group were similar to those in the Noise group. The nuclear arrangement and cellular integrity of OHCs and their relationship to supporting cells was near normal in appearance. Fewer morphological changes were observed in the IHCs in comparison with the Noise group (Figure 4D). Morphological changes in the middle and apical turns were similar to those in other groups.

## Discussion

The results of this study illustrate the extent of ongoing degeneration of SGCs due to noise exposure. The NAC treatment enhanced the survival of SGCs and precluded noise-induced cochlear damage.

The findings of the present study are in agreement with previous studies that confirmed the reduction of SGC density and histological changes in the basal half of the cochlea (3, 7). The reduction in SGC density after noise exposure can be explained by a number of factors. Exposure to noise may cause extensive release of the neurotransmitter glutamate at the IHC synapses, leading to excitotoxicity with a connection loss in the IHC synapses and auditory nerves. Overloading of Ca^++^ following excitotoxicity can damage SGCs (1). Furthermore, acoustic overstimulation causes a reduction in the expression of the AMPA receptor as a glutamate receptor, deletion of the receptors of the GABA neurotransmitter GABA_B1_ in ganglion cells, and changes in cochlear sensitivity to noise (1).

According to our results, SGC density in the Noise group was noticeably reduced in the basal turn, especially in the lower basal turn; this is in contrast to the findings of Lu et al. (6). The difference may be explained by differences in the duration of exposure (10 days versus 1 h) and the type of noise (high-pass white noise centred at 8 kHz versus octave band noise at 10–20 kHz).

Some previous studies have indicated that the base of the cochlea has a greater susceptibility to noise trauma than the apex (29). The following theories may explain the higher levels of qualitative and quantitative damage in the basal region. First, oxidative stress and mitochondrial destruction due to noise exposure cause a higher overload of Ca^++^ in the OHCs within the basal region; additionally, the level of the anti-oxidant glutathione is lower in basal OHCs than in apical OHCs (14, 29). Second, damaged hair cells may survive through self-repair in the apical region, but they may die in the basal turn (30). Third, the immediate defence mechanism against noise exposure causes uncoupling of the TM and OHCs in the middle turn and apex, something that does not occur in the basal turn with its relatively small OC (30, 31).

Histological examination in the lower basal turn showed an increase in basilar membrane thickness, small and pyknotic OHC nuclei, reduction of OHC height, disorganised architecture of OHCs and supporting cells, nuclear stratification of OHCs, and cellular displacement of hair cells and supporting elements. The thickened basilar membrane observed in the Noise group has also been reported in a previous study (32). Furthermore, there is evidence of a partial collapse of the supporting cells towards the basilar membrane, leading to height reduction in the OC (31). In this study, exposure to continuous noise led only to a disarrangement of the outer hair and support cells in the lower basal turn; morphological changes in other structures of the cochlea were not observed. According to a previous study in mice, proper formation of the tunnel of Corti and preservation of the IHCs and OHCs (as well as the supporting cells) were more apparent at the basal than at the apical turn (33). Furthermore, a study using scanning electron microscopy detected disarray of OHC cells in terms of the W/V pattern in the middle and apical turns (34).

The number of hair cells with no intact IHCs or a slight disappearance of IHCs was observed in this study, which is in line with other studies reporting no loss of IHCs (3, 14). Another study reported degenerative changes in the SGCs without ultra-structural changes in the IHCs after exposure to the ototoxic drug carboplatin as an inducer of oxidative stress (35). A number of hypotheses may explain why the SGCs are damaged without severe damage or loss of IHCs. First, neuronal loss may be either the direct effect of the trauma on the sensory neurons or its indirect effect on other ear cells, including supporting cells (17). Secondly, the ErbB receptor signalling is alternated in supporting cells, leading to a reduction in the expression of neurotrophic factor NT-3 with excessive SGC degeneration, with no effect on the appearance or the number of either hair cells or supporting cells (1, 8, 17).

In the present study, administration of NAC promoted SGC density and reduced disorganisation in the architecture of OHCs and supporting cells after exposure to continuous noise, especially in the lower basal turn. Contrary to similar previous studies (12), NAC was here found to have a protective effect on hearing against sub-chronic continuous noise exposure in rats. The mechanism responsible for this protection effect is not fully understood. However, the available evidence implies that NIHL results from enhanced flux of ROS and RNS (such as nitrite oxide), which regulate the survival of hair cells and spiral ganglion neurons in the auditory portion of the inner ear (36–38). High levels of ROS and RNS result in glutathione (GSH) antioxidant compound and glutamate excitotoxicity (4), leading to an imbalance between free radicals and intrinsic antioxidant defences. There is evidence supporting the role of NAC as a free radical scavenger, a substrate for GSH production, a mitochondrial protectant, a glutamate excitotoxicity inhibitor, a lipid peroxidation inhibitor, and a necrosis inhibitor both in OHCs and in dendrites of the afferent neurons (10, 11, 18, 36). Moreover, NAC negatively modulates the production of nitric oxide in endotoxin-treated rats, playing a critical role in neurite outgrowth of neuroblastoma cells (39, 40). An in vitro study reported a protective effect of NAC against cisplatin-induced ototoxicity in both hair cells and auditory nerves (41). There is also evidence that NAC elevates the level of antioxidant glutathione in the basal OHCs more than in the apical OHCs (29). As NAC has shown promise in treating certain diseases in human studies (42, 43), it should be investigated further in the context of the prevention of permanent SNHL.

## Conclusion

NAC offered partial protection against noise exposure. This indicates that oxygen free radicals are not the only mechanism of hearing impairment following acoustic trauma. The administration of NAC is therefore suggested in conjunction with other antioxidants to protect the auditory system through different mechanisms with synergic or additive effects.

## Figures and Tables

**Table 1:** Comparison of mean density between all groups in terms of turns

Turns	Groups	*n*	Density (cell/10000μm^2^) Mean (SD)	*F-*statistics (df1, df2)^a^	*P-*value^a^
Lower Basal	Sham	6	28.28 (5.72)	21.32 (3, 20)	< 0.001^b^
	NAC	6	28.21 (3.20)		
	Noise	6	12.78 (3.57)		
	NoiseNAC	6	17.76 (3.48)		
Upper Basal	Sham	6	20.65 (3.62)	2.64 (3, 20)	0.077
	NAC	6	20.57 (2.70)		
	Noise	6	17.07 (1.52)		
	NoiseNAC	6	19.04 (1.70)		
Lower middle	Sham	6	24.31 (3.10)	2.09 (3, 20)	0.134
	NAC	6	23.33 (3.47)		
	Noise	6	19.11 (2.29)		
	NoiseNAC	6	22.35 (5.62)		
Upper middle	Sham	6	25.8 (5.10)	3.08 (3, 20)	0.051
	NAC	6	24.30 (6.10)		
	Noise	6	19.26 (1.00)		
	NoiseNAC	6	21.66 (0.95)		

a One way ANOVA,

b According to post-hoc Tukey HSD test, no significant difference between the Sham and NAC groups (*P* = 0.98) was found.

There was a significant difference between the Sham and NAC with Noise and NoiseNAC (*P* < 0.001). The Noise and NoiseNAC groups were also significantly different (*P* = 0.046)
